# Study of pain perception under negative emotions and its large-scale brain network dynamics in adolescents with non-suicidal self-injury

**DOI:** 10.3389/fpsyt.2025.1582971

**Published:** 2025-06-05

**Authors:** Yang Zhou, Dongdong Zhou, Zhengyong Zhang, Huiyu Jia, Fang Chen, Liuyi Ran, Xiaorong Chen, Liyang Wan, Yijia Wang, Wo Wang

**Affiliations:** Mental Health Center, University-Town Hospital of Chongqing Medical University, Chongqing, China

**Keywords:** non-suicidal self-injury (NSSI), adolescent, cold pressor test (CPT), EEG microstates, large-scale brain network dynamics

## Abstract

**Background:**

Previous studies have indicated that negative emotions are one of the primary causes of non-suicidal self-injury (NSSI) behavior. This study focuses on examining the characteristics of large-scale brain network dynamics associated with pain perception in NSSI adolescents following experiences of negative emotions.

**Methods:**

A total of 44 adolescents with Major Depressive Disorder (MDD) and NSSI (MDD+NSSI group), 21 MDD adolescents without NSSI (MDD group), and 25 healthy controls (HC group) were recruited. Two emotional conditions (neutral and negative) were established, followed by the simulation of pain conditions using ice water stimulation, while electroencephalogram (EEG) signals were recorded using a 64-channel EEG system. Statistical analysis was conducted using mixed-design repeated measures analysis of variance, with multiple comparisons corrected using the Bonferroni method.

**Results:**

In the MDD+NSSI group, after sadness induction, the duration, time coverage of microstate A, and the transition rate from microstate B→A during the Cold Pressor Test (CPT) were significantly higher compared to neutral emotion induction. Conversely, the occurrence frequency of microstate B decreased markedly. Under neutral emotion induction, the MDD group exhibited higher occurrence frequency of microstate A and transition rate from microstate D→A than the HC group. In contrast, the occurrence frequency, coverage of microstate B, and transition probabilities from microstates C/D→B were significantly lower in the MDD group than in the HC group.

**Conclusion:**

Our findings suggest that adolescents with MDD who exhibit NSSI behavior display abnormalities in large-scale brain network dynamics associated with pain perception following experiences of negative emotions, indicating that EEG microstates may serve as neurobiological markers for abnormal pain perception in NSSI adolescents.

## 1 Introduction

NSSI is defined in the Diagnostic and Statistical Manual of Mental Disorders (DSM-5) as deliberate, repeated, and direct damage to one’s own bodily tissue without suicidal intent, encompassing behaviors such as cutting, burning, hitting, needling, and excessive rubbing, among others (1). Research indicates that adolescent self-injury frequently occurs following the experience of negative emotions, suggesting that NSSI may serve as an emotion regulation strategy to alleviate such states (2, 3). Clinically, many NSSI patients report minimal or absent pain during self-injury, implicating aberrant pain perception in this population (4, 5). Previous studies have typically investigated pain under experimental conditions using thermal, cold, pressure, or electrical stimuli (6). Previous studies have shown that patients with NSSI have abnormal pain perception (7). Borderline Personality Disorder (BPD) patients with NSSI had significantly lower pain scores and significantly higher laser pain thresholds than healthy controls (8). Studies have reported higher cold pain thresholds during CPT in individuals with NSSI than in healthy controls (9). Similarly, reduced pain sensitivity was observed in self-injuring individuals compared to non-injuring controls when pressure was applied to the tibia (10). However, whether such pain perception abnormalities in NSSI patients are specific to negative emotional states or persist under neutral conditions remains unexplored. These critical scientific questions warrant further investigation.

EEG is a convenient and non-invasive tool for recording brain electrical activity. Traditional EEG analyses (e.g., power spectrum and event-related potential analyses) often focus on linear features of specific brain regions or frequency bands. In contrast, microstate analysis captures instantaneous whole-brain neural activity dynamics at a millisecond temporal resolution and quantifies brain state transition patterns using time-series features. Microstate analysis identifies signal peak points via global field power (GFP) and generates microstates representing whole-brain coordinated activity using clustering algorithms, reducing the over-reliance on single channels or local regions seen in traditional methods. It extracts stable scalp potential topography maps through clustering algorithms. These maps, known as microstates, remain stable for 80–120 milliseconds and are referred to as the “atoms of thought” (11). Classical microstates include A, B, C, and D, which together account for 65–84% of the global signal variance (12). World-wide researchers have uniformly reported these four classic microstates, exhibiting high retest consistency (13). Microstate classes (A–D) correspond to resting-state functional MRI (fMRI) networks (e.g., the default mode network and attention networks). EEG microstates reflect large-scale synchronized neural activity across the entire brain. Thus, microstates can represent large-scale brain networks (12), supporting their role as a bridge in cross-modal research. Several studies have employed EEG to investigate neuroelectrophysiological responses associated with pain perception in patients with NSSI. Reduced amplitude of the N2 component in laser-evoked potentials (LEPs) and abnormal conditioned pain modulation (CPM) were observed in patients with NSSI (7). Compared with the MDD and HC groups, patients with BPD who do not feel pain during self-harm demonstrate a significantly increased total absolute theta power (14). Another study observed normal (N1, P2, P3) or moderately enhanced (N2) LEPs amplitudes in BPD patients, proposing that pain attenuation during self-injury in BPD arises from altered intracortical processing resembling meditative states (8).

Although these studies have demonstrated abnormalities in pain perception-related neural responses in individuals with NSSI, they have not explored the neural responses associated with pain perception following negative emotional states. In pain research, various pain induction methods are used. The cold pressor test is the most common experimental pain induction technique in pediatric pain studies and is often used for physiological conditions. We introduced it into adolescent NSSI research. Previous studies have not included emotional conditions, but in this study, pain induction was conducted after emotional induction, which significantly enhances the ecological validity of the study. As previously mentioned, NSSI behavior serves as a strategy for regulating negative emotions and typically occurs subsequent to the experience of such emotional states. Therefore, do individuals with NSSI exhibit abnormalities in the neural responses underlying pain perception under negative emotional conditions? Furthermore, do these responses differ from those observed under neutral emotional states? These questions remain to be further investigated. We hypothesize that individuals with NSSI exhibit abnormalities in pain perception following negative emotional experiences, accompanied by aberrant large-scale brain network dynamics during pain processing. To test this hypothesis, we will experimentally induce neutral and negative emotional states, followed by pain induction via CPT, thereby simulating the real-world phenomenon of self-injury subsequent to negative emotions, to investigate the aforementioned scientific questions.

## 2 Methods

### 2.1 Participants

This study was approved by the Ethics Committee of the University Town Hospital Affiliated to Chongqing Medical University (Approval No.: LL-202363). All participants and their legal guardians involved in the research were fully informed of the research objectives and provided signatures on written informed consent forms.

This study recruited adolescent MDD patients aged 12–17 years who visited the outpatient department or were hospitalized at the Mental Health Centre of University-Town Hospital affiliated with Chongqing Medical University between September 2023 and November 2024 as study participants. Inclusion criteria: ① Meeting the diagnostic criteria for MDD in DSM-5 or the criteria for “depressive episode” in International Classification of Diseases(ICD-10); ② Total score of the 17-item Hamilton Depression Rating Scale (HAMD-17) ≥17 at enrolment; ③ Right-handedness. Exclusion criteria: ① Comorbid severe cardiac, hepatic, or renal dysfunction; ② Comorbid endocrine, hematological, or autoimmune diseases; ③ History of cerebral organic diseases, head trauma, epilepsy, or other central nervous system disorders; ④ History of other severe psychiatric disorders; ⑤ Alcohol abuse or use of other psychoactive substances. Age- and gender-matched healthy controls were also recruited. According to the definition of NSSI frequency criteria in the DSM-5, the exclusion criteria for the MDD+NSSI group were: no NSSI behavior or NSSI episodes did not exceed 5 days in total. The exclusion criteria for MDD group were: one or more episodes of NSSI. The demographic and clinical characteristics of the study participants are presented in **Table 1**. The Hamilton Rating Scale for Depression-17 (HAMD) was used to assess the severity of depression.

**Table 1:** Demographic and clinical characteristics.

Demographic	HC	MDD	MDD+NSSI	*P*
Sample size	25	21	44	
Age(years),mean ± SD	14.96 ± 1.27	15.19 ± 1.17	14.59 ± 1.15	0.141
Gender (Male/Female)	11/14	5/16	11/33	0.198
BMI(kg/m²),mean ± SD	19.96 ± 4.08	18.63 ± 2.33	20.26 ± 2.93	0.150
Disease Duration(month),mean ± SD	–	10.38 ± 6.11	15.41 ± 8.64	0.020
HAMD-17,mean ± SD	–	20.38 ± 2.85	23.86 ± 4.38	0.002
self-harm(frequency),mean ± SD	–	–	46.55 ± 52.37	–
Drug therapy	–	SSRIs	SSRIs	–

HC, healthy control group; MDD, Adolescents with MDD without NSSI; MDD+NSSI, Adolescents with MDD with NSSI; HAMD-17, Hamilton Depression Scale-17.

### 2.2 Procedure of CPT

Previous studies have indicated that the CPT is the most commonly used experimental pain induction technique in pediatric pain research. The CPT involves immersing the hand or forearm in cold water (typically 10°C) to elicit mild to moderate pain (15). Recent studies have recommended using colder water temperatures (5–7°C) for children aged 8 years and above, as opposed to the 10°C suggested in the 2005 guidelines (16). Therefore, this study used 5°C water as the pain-stimulus condition. A thermostatic water bath was used to maintain the water temperature at ±0.5°C of the target temperature. The left hand was subjected to the cold pressor test in 5°C water, while the right hand was placed in 25°C water to control for variables, thereby enhancing the accuracy of pain measurement.

Step 1 (Neutral Emotion Induction): Assess participants using the Positive and Negative Affect Schedule (PANAS); induce neutral emotion; reassess using PANAS. Step 2 (CPT): ① Immerse left hand in 5°C water bath, right hand in 25°C water. ② Participants report pain, and researchers record via button press in the EEG paradigm. ③ If the pain becomes intolerable for the participant or the maximum set time of 3 minutes is reached, remove both hands. Researchers then press a button in the EEG paradigm to record this. ④ The participant’s hands were removed and then the Faces Pain Scale-Revised (17) pain rating was conducted. Step 3: The participant rested fully until the pain sensation disappeared and their emotions stabilized. Step 4 (Negative Emotion Induction): Assess participants using PANAS; induce negative emotion; reassess using PANAS. Step 5 (CPT): Same as step 2.

**Figure 1:**
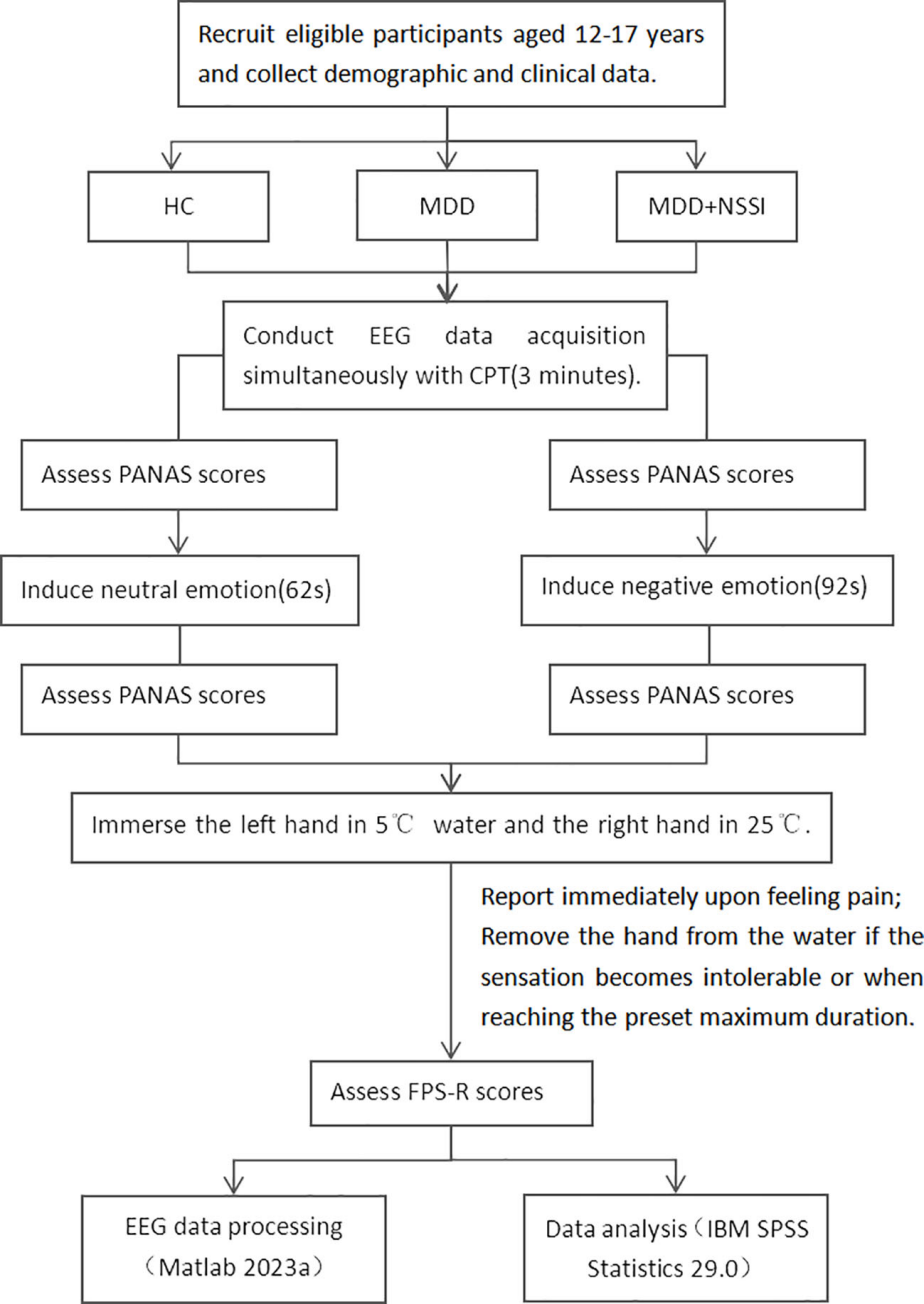
Experimental process.

Given that the subjects are MDD patients, who experience marked emotional fluctuations, low energy, and a longer time to recover calm emotions, it’s essential to prioritize completing all experimental procedures when their emotions are relatively stable. Thus, in our experimental process, neutral mood induction is conducted first, followed by negative mood induction. All scales used in the experimental process mentioned above are widely recognized for their good reliability and validity. PANAS is used to measure the participants’ immediate emotional responses during the experiment, and its NA (Negative Affect) subscale evaluates the effectiveness of negative emotion induction. FPS-R is applied for pain assessment in adolescent participants. The videos for emotion induction were film clips, which are widely used in emotion research for their high ecological validity. This study used clips from a standardized database of Chinese emotional films, where each clip is designed to elicit only one specific emotion. A 92-second clip from 《Changjiang Qihao》 was used to induce sadness, while a 65-second clip from 《Black Coal, Thin Ice》 was used for neutral emotion induction (18). The experimental process is shown in **Figure 1**.

### 2.3 EEG data acquisition

In this study, EEG data were collected using a 64-channel scalp electrode acquisition system (Neuroscan) compliant with the international 10–20 system.

Data acquisition was conducted in a quiet, well-lit room. Participants were instructed to fixate on a screen positioned directly ahead, maintain a relaxed seated posture, minimize bodily movements, and blink naturally. The sampling rate is set to 1000Hz, and the resistance of each channel is required to be below 15kΩ. EEG data were collected from each participant under two experimental conditions. Condition 1: CPT was performed after viewing neutral emotional video material; Condition 2: CPT was performed after viewing sad video material.

### 2.4 EEG data preprocessing

The preprocessing of EEG data was performed using the EEGLAB (version 2023.0) toolkit based on MATLAB 2023a. Following data import, a 1–40 Hz bandpass filtering was applied. After removing irrelevant electrodes, bad segments were deleted and bad channels (if present) were interpolated. Ocular and electromyographic artefacts were removed via Independent Component Analysis (ICA). Finally, average re-referencing across the entire brain was conducted.

### 2.5 EEG microstate analysis

The microstate analysis in this study was conducted using the Microstate 1.0 plugin based on EEGLAB (19). Initially, band-pass filtering between 8 and 13Hz was performed. A group-level template was constructed by randomly selecting 1000 Global Field Power (GFP) peaks from each participant. The modified k-means algorithm was employed to cluster microstates in the group-level template derived from GFP peaks, with the number of microstate templates set to 4. Subsequently, the template microstates were back-fitted to each raw EEG recording, followed by temporal smoothing (smoothing parameter = 30 ms). Finally, Global Explained Variance (GEV) and microstate dynamic metrics were calculated for each participant. The microstate dynamic metrics included duration, coverage, occurrence, and transition probabilities between microstates. Microstate temporal parameters (duration, occurrence, coverage) and transition probability are key indicators in EEG microstate analysis, reflecting the dynamic characteristics and functional integration capabilities of brain neural networks (12). Duration refers to the average length of time a single microstate persists (in milliseconds), reflecting the ability of specific neural clusters to maintain a stable state, that is, the average time a set of neural generators maintain synchronized activity. Occurrence frequency refers to the average number of times each microstate appears per unit time, representing the tendency of a set of neural generators to coordinate their activities over time. The higher the occurrence frequency, the more frequently the corresponding brain network is activated. Coverage refers to the proportion of time a particular type of microstate occupies of the total time. Coverage reflects the relative importance of the microstate throughout the recording period. Transition probability refers to the probability of switching between different microstates, reflecting the dynamic interaction patterns between different brain networks.

### 2.6 Statistical analysis

Statistical analyses were performed using IBM SPSS Statistics 29.0. All data analysis followed the same principle: parametric tests for normally or approximately normally distributed data, and non-parametric tests for skewed data. A chi-square test was employed to evaluate gender distribution differences among the three groups. A one-way analysis of variance (ANOVA) was utilized to assess differences in age and BMI across groups. An independent-sample t-test was conducted to evaluate the differences in disease duration and HAMD-17 scores between the two groups. A paired-samples t-test was used to evaluate the differences in NA scores before and after mood induction among the participants. A repeated-measures ANOVA was applied to examine the differences among three groups in pain ratings, pain tolerance, and microstate dynamics indicators under two conditions. Generalized estimating equations were used for statistical analysis because pain thresholds and the durations of microstates A/B were not normally distributed. Within-subject variables included emotional categories (neutral vs. sad emotions), while between-subject variables comprised diagnostic groups (HC, MDD, MDD+NSSI). Results of main effects and simple effects analyses are presented in tabular format. For *post hoc* comparisons, Bonferroni correction was implemented to adjust p-values. Spearman’s correlation analysis was conducted to investigate associations between microstate dynamics metrics and pain thresholds/tolerance.

## 3 Results

### 3.1 Efficacy of negative emotion induction

NA scores across all three groups showed statistically significant differences before and after negative emotion induction, confirming successful elicitation of the targeted affective state. Results are presented in **Table 2**.

**Table 2:** Efficacy of negative emotion induction.

Variables	HC	MDD	MDD+NSSI
Pre-NA	12.44 ± 4.24	14.71 ± 6.38	15.98 ± 6.60
Post-NA	14.92 ± 5.33	17.33 ± 6.52	18.89 ± 6.64
*T*	2.379	2.215	3.034
*P*	0.026*	0.038*	0.004*

HC, healthy control group; MDD, Adolescents with MDD without NSSI; MDD+NSSI, Adolescents with MDD with NSSI; Pre-NA, Pre-negative emotion induction NA scores; Post-NA, Post-negative emotion induction NA scores, *p < 0.05.

### 3.2 Pain indicators

The present study found that the pain threshold under negative emotional states in the HC group was significantly higher than under neutral states. Compared to neutral states, the MDD+NSSI group exhibited significantly increased pain thresholds and pain tolerance, alongside significantly reduced FPS-R scores under negative emotional conditions. The results are summarized in **Table 3**.

**Table 3:** Pain indicators.

Pain indicators	Variables	HC	MDD	MDD+NSSI	*F/H*	*P*
FPS-R score	Neutral	6.36 ± 1.52	5.95 ± 1.88	5.86 ± 2.21	0.523	0.595
	Negative	6.32 ± 1.74	6.52 ± 2.29	5.30 ± 2.32	3.017	0.054
	*F*	0.017	2.999	6.218		
	*P*	0.895	0.087	0.015*		
	Main effect (*F*,*P*)	0.005,0.942				
	Intergroup effect (*F*,*P*)	1.549,0.218				
	Interaction effect (*F*,*P*)	4.139,0.019*				
Pain threshold	Neutral	12.17 (8.90,20.14)	15.9 (9.56,19.98)	17.08 (10.62,25.09)	2.501	0.286
	Negative	18.59 (11.97,32.52)	13.96 (9.63,27.09)	18.08 (11.10,37.05)	1.259	0.533
	*Z*	−2.354	−1.547	−2.101		
	*P*	0.019*	0.122	0.036*		
	Main effect (*χ²*,*P*)	10.777,0.001*				
	Intergroup effect (*χ²*,*P*)	1.793,0.408				
	Interaction effect (*χ²*,*P*)	3.144,0.208				
Pain tolerance	Neutral	95.96 ± 70.50	86.03 ± 81.23	97.27 ± 81.90	0.152	0.859
	Negative	107.12 ± 88.10	100.45 ± 90.72	126.16 ± 97.57	0.657	0.521
	*F*	1.299	1.819	15.315		
	*P*	0.258	0.181	<0.001*		
	Main effect (*F*,*P*)	11.211,0.001*				
	Intergroup effect (*F*,*P*)	1.262,0.288				
	Interaction effect (*F*,*P*)	1.262,0.288				

HC, healthy control group; MDD, Adolescents with MDD without NSSI; MDD+NSSI, Adolescents with MDD with NSSI, *p < 0.05.

### 3.3 Microstate analysis

**Figure 2:**
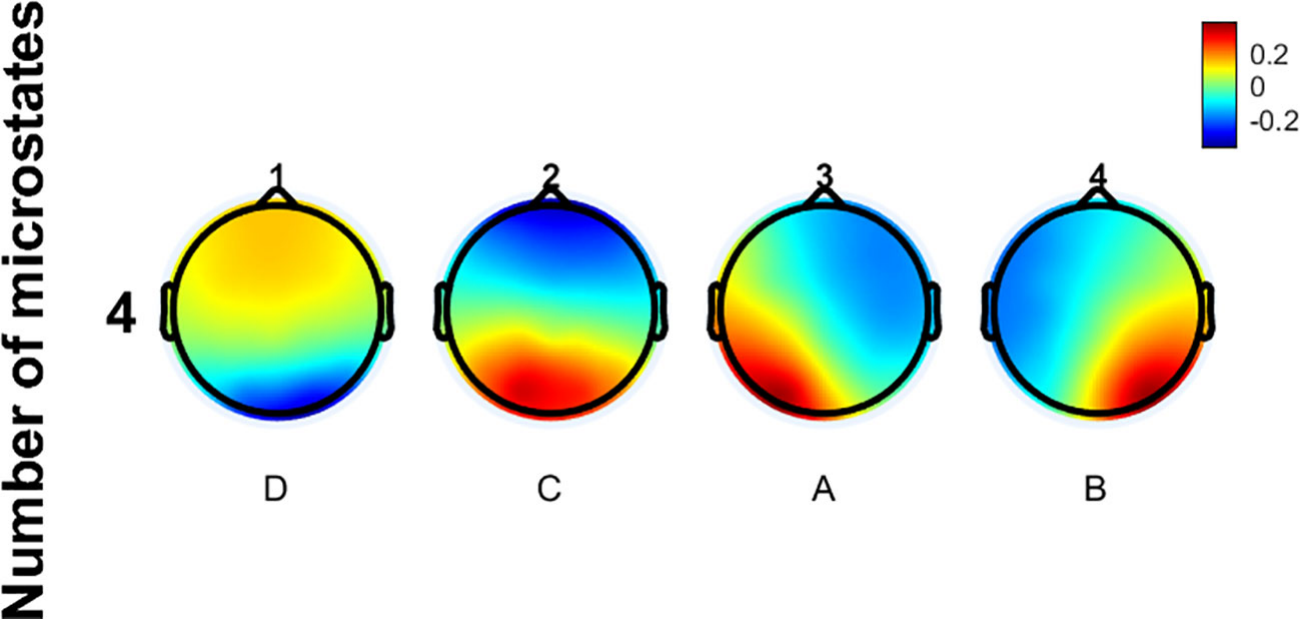
The micro-state topographic map of all subjects.

The EEG microstate data of all participants in this study were clustered, revealing four prototypical microstates as shown in **Figure 2**. The microstate classes corresponding to the topographic maps in **Figure 2** are D, C, A, and B. Their topological structures are similar to those of the four classic microstates previously reported. Microstate A has its positive and negative poles located in the right frontal and left occipital regions. For Microstate B, they are in the left frontal and right occipital regions. Microstate C’s poles are in the frontal and occipital regions. Microstate D’s poles are in the frontocentral and occipital regions. No significant differences in GEV were observed among the three groups.

#### 3.3.1 Microstate dynamics parameters

All temporal parameters of microstates (duration, coverage, and occurrence) were analyzed using repeated-measures ANOVA with Bonferroni correction for *post hoc* comparisons. The results are presented in **Table 4**.

**Table 4:** Three groups of EEG microstate dynamics.

Microstate dynamics	Variables	HC	MDD	MDD+NSSI	*F*	*P*	Main effect (*F*/*χ²*,*P*)	Intergroup effect (*F*/*χ²*,*P*)	Interaction effect (*F*/*χ²*,*P*)
Dur.A	Neutral	115.48 (110.63,127.44)	128.27 (112.53,142.76)	126.79 (109.85,148.11)	3.679	0.159	5.644,0.018	6.195,0.045	0.994,0.608
	Negative	120.15 (109.78,136.75)	123.26 (108.60,146.55)	137.50 (111.42,158.39)	3.072	0.215			
	*Z*	−1.063	−0.122	−2.147					
	*P*	0.288	0.903	0.032					
Dur.B	Neutral	131.12 (120.59,147.06)	115.65 (102.28,132.39)a	125.04 (105.05,140.94)	6.484	0.039	0.282.0.595	7.95,0.019	0.039,0.981
	Negative	131.10 (119.09,140.87)	117.34 (101.94,133.56)	118.88 (104.41,144.83)	5.82	0.054			
	*Z*	−0.417	−0.122	−0.735					
	*P*	0.677	0.903	0.462					
Dur.C	Neutral	129.69 ± 15.09	124.38 ± 15.39	128.13 ± 26.21	0.371	0.691	0.034,0.854	0.544,0.582	0.063,0.939
	Negative	130.85 ± 19.65	124.75 ± 17.55	127.71 ± 20.87	0.544	0.582			
	*Z*	0.103	0.009	0.024					
	*P*	0.749	0.925	0.876					
Dur.D	Neutral	117.81 ± 15.45	126.85 ± 26.52	123.23 ± 19.60	1.161	0.318	0.003,0.960	1.063,0.350	0.317,0.729
	Negative	119.45 ± 13.93	124.56 ± 22.90	123.60 ± 16.83	0.592	0.555			
	*Z*	0.233	0.383	0.021					
	*P*	0.631	0.538	0.885					
Cov.A	Neutral	0.21 ± 0.06	0.27 ± 0.09	0.25 ± 0.10	2.974	0.056	3.886,0.052	2.845,0.064	1.059,0.351
	Negative	0.22 ± 0.07	0.27 ± 0.15	0.28 ± 0.11	2.354	0.101			
	*Z*	0.977	0.04	7.531					
	*P*	0.326	0.843	0.007					
Cov.B	Neutral	0.29 ± 0.08	0.21 ± 0.09a	0.25 ± 0.11	3.081	0.051	1.582,0.212	3.290,0.042	0.219,0.804
	Negative	0.28 ± 0.09	0.21 ± 0.09	0.23 ± 0.11	2.54	0.085			
	*Z*	0.668	0.033	2.03					
	*P*	0.416	0.857	0.158					
Cov.C	Neutral	0.26 ± 0.06	0.24 ± 0.05	0.25 ± 0.08	0.42	0.659	0.047,0.828	0.596,0.553	0.903,0.409
	Negative	0.26 ± 0.07	0.25 ± 0.07	0.24 ± 0.08	0.854	0.429			
	*Z*	0.000	0.362	1.878					
	*P*	0.988	0.549	0.174					
Cov.D	Neutral	0.22 ± 0.05	0.25 ± 0.07	0.23 ± 0.06	1.599	0.208	0.475,0.493	1.656,0.197	0.112,0.894
	Negative	0.22 ± 0.04	0.24 ± 0.06	0.23 ± 0.06	1.105	0.336			
	*Z*	0.040	0.5	0.052					
	*P*	0.842	0.481	0.82					
Occ.A	Neutral	1.72 ± 0.26	1.99 ± 0.26a	1.86 ± 0.39	3.558	0.033	0.003,0.957	2.934,0.058	3.841,0.025
	Negative	1.77 ± 0.30	1.85 ± 0.29	1.95 ± 0.31	2.682	0.074			
	*Z*	0.573	4.199	3.398					
	*P*	0.451	0.043	0.069					
Occ.B	Neutral	2.07 ± 0.25	1.76 ± 0.46a	1.85 ± 0.46	3.476	0.035	3.181,0.078	3.955,0.023	0.435,0.649
	Negative	2.00 ± 0.24	1.74 ± 0.46	1.74 ± 0.48	3.261	0.043			
	*Z*	1.120	0.095	4.322					
	*P*	0.293	0.758	0.041					
Occ.C	Neutral	2.05 ± 0.33	1.99 ± 0.28	1.96 ± 0.37	0.475	0.623	0.696,0.406	1.062,0.350	1.084,0.343
	Negative	2.02 ± 0.37	2.01 ± 0.43	1.88 ± 0.38	1.515	0.226			
	*Z*	0.151	0.166	3.662					
	*P*	0.699	0.684	0.059					
Occ.D	Neutral	1.90 ± 0.26	2.00 ± 0.31	1.89 ± 0.28	1.08	0.344	0.844,0.361	1.317,0.273	0.030,0.971
	Negative	1.86 ± 0.26	1.97 ± 0.34	1.87 ± 0.31	0.966	0.385			
	*Z*	0.452	0.2	0.236					
	*P*	0.503	0.656	0.629					

HC, healthy control group; MDD, Adolescents with MDD without NSSI; MDD+NSSI, Adolescents with MDD with NSSI; a means that compared with HC group*p < 0.05, Dur, Duration; Cov, Coverage; Occ, Occurrence; A, microstate A; B, microstate B; C, microstate C; D, microstate D.

For microstate A, within the NSSI group, the duration/coverage of microstate A during CPT following sad emotion induction was higher compared to that following neutral emotion induction. The interaction effect on the occurrence of microstate A was significant. Simple effect analysis revealed that during the CPT following neutral emotion induction, the occurrence of microstate A in the MDD group was higher than that in the HC group. Within the MDD group, the occurrence of microstate A during the CPT following sad emotion induction was lower than that following neutral emotion induction.

**Figure 3:**
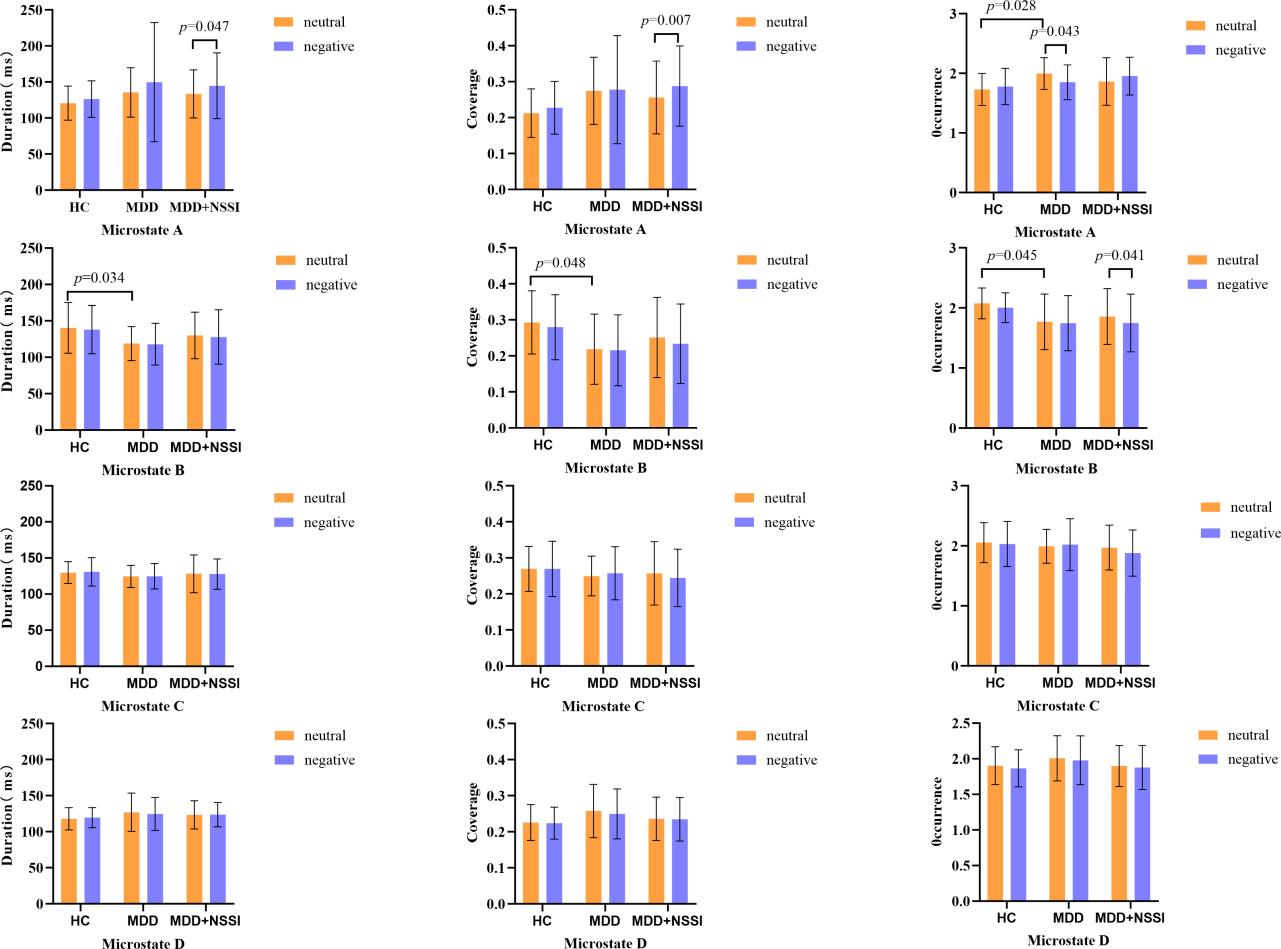
Histogram of microstate dynamics. HC, healthy control group; MDD, Adolescents with MDD without NSSI; MDD+NSSI, Adolescents with MDD with NSSI. Neutral: CPT after neutral emotion induction; Negative: CPT after negative emotion induction.

For microstate B, within the MDD+NSSI group, the occurrence of microstate B during the CPT following sad emotion induction was lower than that following neutral emotion induction. During the CPT following neutral emotion induction, the occurrence, duration, and coverage of microstate B in the MDD group were lower than those in the HC group. The results are summarized in **Figure 3**.

#### 3.3.2 Microstate transition probabilities

**Table 5** displays the microstate transition probabilities among the three groups. For transition probabilities, within the MDD group, the transition rate of microstate D→A during CPT after sad emotion induction was lower than that after neutral emotion induction. Within the MDD+NSSI group, the transition rate of microstate B→A during CPT after sad emotion induction was higher than that after neutral emotion induction. Conversely, the transition rates of microstate B→C and C→B during CPT after sad emotion induction were lower than those after neutral emotion induction. During CPT under neutral emotion induction, the MDD group exhibited a lower transition rate of microstate C→B compared to the HC group; the MDD group showed a higher transition rate of microstate D→A than the HC group; both MDD and MDD+NSSI groups demonstrated lower transition rates of microstate D→B compared to the HC group. During CPT under sad emotion induction, the MDD+NSSI group displayed higher transition rates of microstate A→D and B→A than the HC group, while the MDD group exhibited a lower transition rate of microstate C→B compared to the HC group.

**Table 5:** Transfer probability between the three groups.

Transfer probability	Variables	HC	MDD	MDD+NSSI	*F*	*P*	Main effect (*F*,*P*)	Intergroup effect (*F*,*P*)	Interaction effect (*F*,*P*)
A→B	Neutral	0.36 ± 0.09	0.32 ± 0.11	0.35 ± 0.11	0.703	0.498	0.036,0.850	1.689,0.191	1.057,0.352
	Negative	0.38 ± 0.08	0.32 ± 0.10	0.33 ± 0.10	2.493	0.089			
	*F*	0.782	0.058	1.543					
	*P*	0.379	0.810	0.217					
A→C	Neutral	0.29 ± 0.05	0.28 ± 0.04	0.28 ± 0.09	0.223	0.8	0.007,0.932	0.323,0.725	0.357,0.701
	Negative	0.28 ± 0.07	0.30 ± 0.08	0.28 ± 0.07	0.44	0.645			
	*F*	0.205	0.469	0.041					
	*P*	0.652	0.495	0.839					
A→D	Neutral	0.33 ± 0.06	0.38 ± 0.09	0.35 ± 0.09	1.417	0.248	0.022,0.881	2.891,0.061	1.588,0.210
	Negative	0.32 ± 0.04	0.37 ± 0.08	0.38 ± 0.08a	4.13	0.019			
	*F*	0.412	0.159	3.092					
	*P*	0.523	0.691	0.082					
B→A	Neutral	0.31 ± 0.06	0.34 ± 0.09	0.35 ± 0.09	1.877	0.159	6.626,0.012	3.707,0.029	1.117,0.332
	Negative	0.32 ± 0.07	0.36 ± 0.13	0.40 ± 0.11a	3.767	0.027			
	*F*	0.461	1.13	10.448					
	*P*	0.499	0.291	0.002					
B→C	Neutral	0.39 ± 0.07	0.38 ± 0.06	0.37 ± 0.09	0.148	0.862	1.541,0.218	1.120,0.331	1.487,0.232
	Negative	0.39 ± 0.08	0.36 ± 0.10	0.35 ± 0.08	2.082	0.131			
	*F*	0.192	0.516	5.184					
	*P*	0.662	0.474	0.025					
B→D	Neutral	0.29 ± 0.07	0.27 ± 0.05	0.26 ± 0.06	1.566	0.215	3.945,0.050	2.327,0.104	0.140,0.870
	Negative	0.27 ± 0.06	0.26 ± 0.06	0.24 ± 0.07	1.675	0.193			
	*F*	1.776	0.364	3.013					
	*P*	0.186	0.548	0.086					
C→A	Neutral	0.24 ± 0.07	0.29 ± 0.07	0.27 ± 0.09	2.338	0.103	2.529,0.115	2.576,0.082	0.270,0.764
	Negative	0.25 ± 0.088	0.29 ± 0.12	0.30 ± 0.08	1.578	0.212			
	*F*	0.983	0.096	3.03					
	*P*	0.324	0.758	0.085					
C→B	Neutral	0.41 ± 0.07	0.32 ± 0.09a	0.36 ± 0.10	5.293	0.007	5.318,0.023	6.514,0.002	0.100,0.905
	Negative	0.39 ± 0.08	0.30 ± 0.09a	0.33 ± 0.10	4.456	0.014			
	*F*	1.754	0.77	4.222					
	*P*	0.189	0.383	0.043					
C→D	Neutral	0.34 ± 0.06	0.38 ± 0.07	0.35 ± 0.06	1.684	0.192	0.721,0.398	2.144,0.123	0.063,0.939
	Negative	0.34 ± 0.07	0.39 ± 0.11	0.36 ± 0.07	1.509	0.227			
	*F*	0.154	0.465	0.132					
	*P*	0.696	0.497	0.717					
D→A	Neutral	0.30 ± 0.06	0.40 ± 0.09a	0.36 ± 0.13	4.854	0.01	0.540,0.465	3.070,0.051	6.329,0.003
	Negative	0.32 ± 0.07	0.35 ± 0.11	0.38 ± 0.11	2.179	0.119			
	*F*	1.209	9.522	1.93					
	*P*	0.275	0.003	0.168					
D→B	Neutral	0.31 ± 0.08	0.23 ± 0.09a	0.25 ± 0.10a	4.839	0.01	0.038,0.846	2.913,0.060	2.783,0.067
	Negative	0.28 ± 0.08	0.26 ± 0.13	0.24 ± 0.10	1.164	0.317			
	*F*	2.659	2.741	0.44					
	*P*	0.107	0.101	0.509					
D→C	Neutral	0.37 ± 0.07	0.36 ± 0.07	0.38 ± 0.09	0.365	0.695	1.196,0.277	0.042,0.959	1.542,0.220
	Negative	0.38 ± 0.07	0.38 ± 0.08	0.37 ± 0.09	0.243	0.785			
	*F*	0.595	2.232	0.601					
	*P*	0.443	0.139	0.44					

HC, healthy control group; MDD, Adolescents with MDD without NSSI; MDD+NSSI, Adolescents with MDD with NSSI; a means that compared with HC group *p < 0.05, A, microstate A; B, microstate B; C, microstate C; D, microstate D.

### 3.4 Correlation analysis

**Figure 4:**
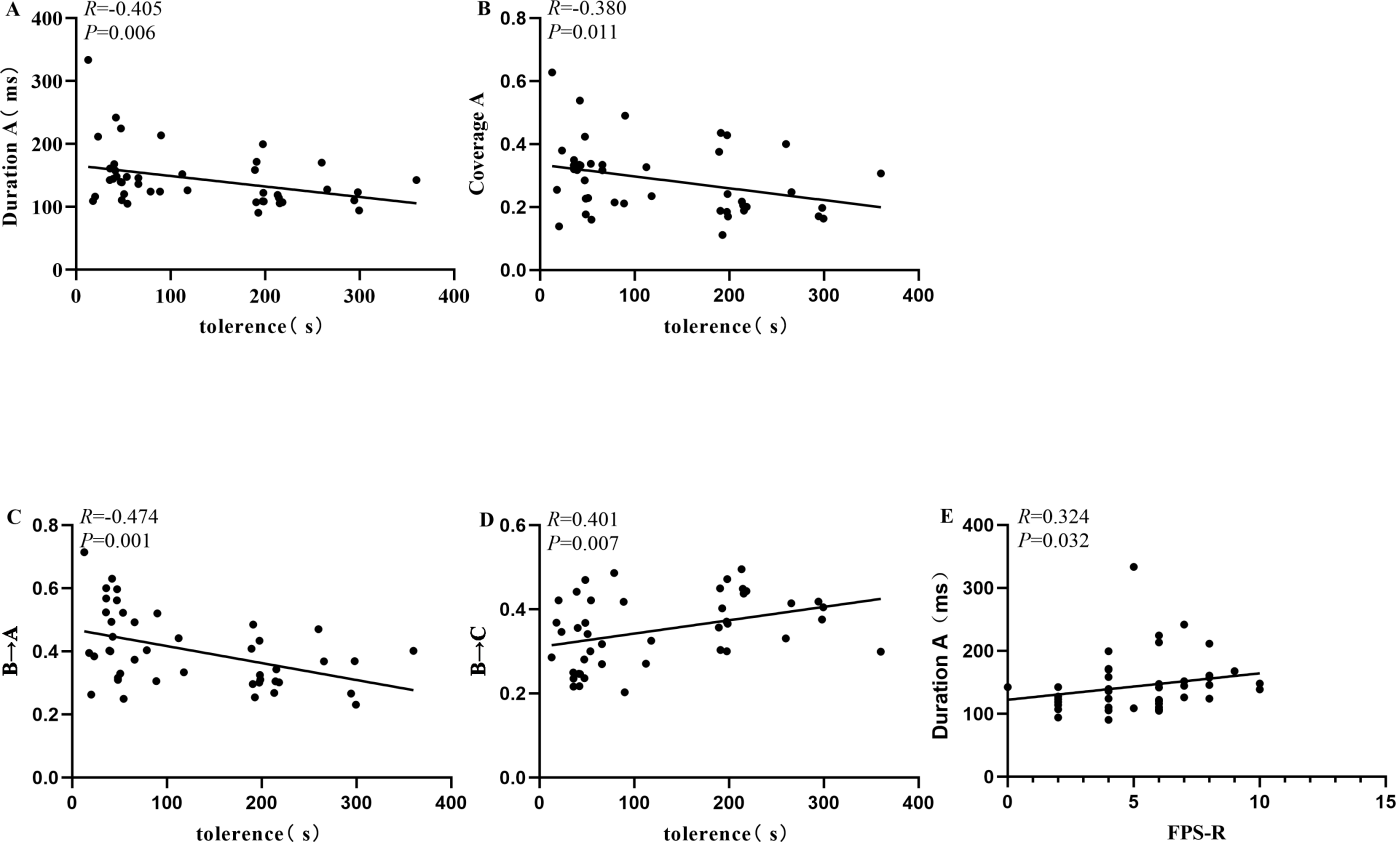
Correlation analysis. **(A)** Correlation analysis of the duration of microstate A with pain tolerance, **(B)** Correlation analysis of the coverage of microstate A with pain tolerance, **(C)** Correlation analysis of the transition rate from microstate B to A with pain tolerance, **(D)** Correlation analysis of the transition rate from microstate B to C with pain tolerance, **(E)** Correlation analysis of the duration of microstate A with FPS-R score.

We conducted a correlation analysis between microstate indicators showing significant differences in the MDD+NSSI group during the CPT after sadness induction and pain tolerance under sad mood. The results are presented in **Table 6**. The duration and coverage of microstate A, as well as the transition rate from B→A, showed negative correlations with pain tolerance. In contrast, the transition rate from B→C exhibited a positive correlation with pain tolerance. Additionally, the duration of microstate A was positively correlated with FPS-R scores. Significant results are visualized in **Figure 4**.

**Table 6:** Correlation analysis.

Variables	Pain tolerance		Pain threshold		FPS-R score	
	*P*	*R*	*P*	*R*	*P*	*R*
Duration A	0.006	−0.405**	0.098	−0.252	0.032	0.324*
Coverage A	0.011	−0.380*	0.087	−0.261	0.103	0.249
Occurrence B	0.434	0.121	0.112	0.243	0.051	−0.296
A→D	0.815	−0.036	0.257	−0.175	0.593	0.083
B→A	0.001	−0.474**	0.062	−0.284	0.072	0.273
B→C	0.007	0.401*	0.318	0.154	0.129	−0.233
C→B	0.659	0.068	0.315	0.155	0.348	−0.145

HC, healthy control group; MDD, Adolescents with MDD without NSSI; MDD+NSSI, Adolescents with MDD with NSSI, **p<0.01 (two-tailed), *p<0.05 level (two-tailed).

## 4 Discussion

Under experimental conditions, we induced negative emotion followed by cold pain stimulation to simulate the post-NSSI phenomenon following negative emotions widely observed in real-world settings. Using EEG microstate analysis, we investigated alterations in large-scale brain network dynamics among adolescents with MDD accompanied by NSSI. We compared behavioral indicators related to pain perception (including pain intensity, pain threshold, and pain tolerance) and EEG microstate dynamics under two emotional conditions (neutral emotion vs. sad emotion) across the HC, MDD, and MDD+NSSI groups. Our findings revealed that the NSSI group exhibited specific behavioral changes in pain perception under negative emotional conditions: compared with neutral emotion conditions, they demonstrated increased pain thresholds, enhanced pain tolerance, and reduced pain intensity after experiencing negative emotions. Furthermore, we identified corresponding alterations in EEG microstate dynamics, which were significantly correlated with behavioral indicators of pain perception. These results suggest abnormal pain perception following negative emotions in NSSI patients, with microstate dynamics potentially serving as objective neurobiological markers for such abnormalities.

Regarding pain indicators, individuals with NSSI exhibited reduced pain perception following exposure to negative emotions. Previous studies have identified abnormal pain perception in NSSI patients (20). Elevated pressure pain thresholds and pressure pain tolerance in NSSI patients have also been demonstrated (21). Adolescents with NSSI exhibited increased pain thresholds and enhanced hypothalamic-pituitary-adrenal (HPA) axis responses to pain stimuli (9). Women with NSSI diagnosed with BPD exhibited increased thermal pain thresholds and reduced pain ratings (8). Previous studies and this study’s pain metrics both indicate abnormal pain perception in individuals with NSSI. This is largely consistent with our hypothesis. Regrettably, our findings are limited to within the MDD+NSSI group. Compared to the MDD group, the MDD+NSSI group showed non-significant reductions in pain ratings and increases in pain threshold and pain tolerance. This may be attributed to the relatively mild severity of self-injury among the adolescent participants. Notably, while the HC group exhibited significantly enhanced pain thresholds post-negative emotion exposure, only the MDD + NSSI group demonstrated substantial changes in pain tolerance and pain intensity following negative emotional induction.

Compared to neutral emotion, the HC and MDD+NSSI groups exhibited significantly elevated pain thresholds under sad emotion. In our experimental protocol, pain thresholds were first measured under neutral emotion followed by sad emotion. Continuous CPT may lead to the occurrence of adaptation phenomena (22). Thus, the observed elevation in pain thresholds in the HC and MDD+NSSI groups could stem from adaptive neurophysiological changes to sequential nociceptive stimuli. However, MDD patients exhibited no significant elevation in pain thresholds. This suggests adaptive dysfunction to continuous pain perception in MDD patients, potentially reflecting MDD-specific cerebral functional deficits. Notably, MDD patients with NSSI not only lacked such adaptive impairments but demonstrated paradoxical hyperadaptation—manifested as significantly enhanced pain thresholds and tolerance alongside reduced pain perception under sad emotion compared to neutral emotion. This phenomenon may partly explain the repetitive NSSI behaviors, as these patients experience diminished pain perception or even derive pleasure from self-injury. Our findings align with studies reporting reduced baseline endorphin levels in NSSI patients, who may activate endogenous opioid systems via NSSI to release endorphins, thereby achieving analgesia, alleviating negative emotion, or inducing euphoria (23–25).

Compared to the neutral emotion, the MDD+NSSI group exhibited significantly increased pain tolerance and decreased FPS-R pain scores under the Sad emotion condition, whereas no such alterations were observed in the HC and MDD groups. This indicates abnormal subjective pain perception in MDD+NSSI patients. Emotional dysregulation plays a direct role in the association between self-injury and pain attenuation (20). The Experiential Avoidance Model posits that NSSI serves as a functional behavior to escape or avoid overwhelming negative emotions; this behavior reinforces its occurrence through immediate emotional relief, thereby forming a vicious cycle (26). The Benefit and Barrier Model suggests that the initiation and maintenance of NSSI involve multiple benefit factors (e.g., emotion regulation, self-punishment, interpersonal communication) alongside barrier factors (e.g., pain perception, aversive reactions). The model emphasizes that NSSI occurs only when individuals overcome these “barriers” to obtain “benefits” (27). In other words, the protective “barrier” of pain perception in MDD+NSSI patients becomes compromised through repeated NSSI behaviors. Our findings of attenuated pain perception provide robust evidence for this mechanism.

These findings elucidate the clinical phenomenon reported by patients with NSSI that NSSI behaviors alleviate Negative emotion and reduce pain perception (28). Concurrently, our EEG study has uncovered evidence related to large-scale brain network dynamics.

We observed that in the MDD+NSSI group during the CPT under Sad emotion induction, the duration and coverage of microstate A were elevated compared to those following neutral emotion induction. Conversely, the occurrence of microstate B exhibited a reduction. Between-group differences were exclusively identified in the MDD cohort: following neutral emotion induction, the MDD group demonstrated higher occurrence of microstate A relative to the HC group during CPT. In contrast, the coverage, occurrence, and duration of microstate B were diminished compared to the HC group.

The activity of microstate A primarily originates from the left temporal lobe and left insula, and is closely associated with anterior cingulate cortex (ACC) activity (29–31). The ACC is implicated in pain processing, with its dorsal region encoding the affective dimension of pain, while the somatosensory cortex (S1, S2) encodes pain intensity (32, 33). The ACC participates in emotional and cognitive control by modulating default mode network (DMN) activity (34, 35), and its dysfunction has been identified in depressive patients (36). The insula, a hub for gustatory perception, interoception, emotion, and decision-making orchestration, plays a pivotal role in integrating sensory, affective, and cognitive information (37). The posterior insula receives nociceptive signals via the spinothalamic pathway, encoding pain intensity and location (sensory dimension) (38). The anterior insula integrates the affective and cognitive components of pain and is strongly linked to socio-emotional experiences (e.g., empathy for pain) (38). The activity of microstate A may relate to the insula’s roles in emotional regulation and interoceptive processing (39). Additionally, studies highlight the synergistic roles of the insula and ACC in emotional processing and pain perception. The insula (particularly anterior) and ACC (especially dorsal) constitute the “pain matrix,” integrating sensory, affective, and cognitive dimensions of pain (40). Dynamic changes in insula-ACC functional connectivity exhibit high correlation with subjective pain intensity reports (41). Different studies have found a link between ACC/insula dysfunction and NSSI. In depression, the whole-brain functional connection between the right insula and other regions is negatively correlated with NSSI frequency over a year (42). In NSSI patients, abnormal ACC activation during cognitive interference tasks may relate to their impaired emotional regulation and impulse control (43). In the MDD+NSSI group, activation of the ACC or insula during sad emotion may simultaneously engage their cognitive and processing functions for pain and emotion. Thus, under equivalent pain stimuli, increased microstate A in the MDD+NSSI group suggests heightened reactivity to sad emotion and attenuated pain perception. This conclusion aligns strongly with FPS-R scores. A recent study concurs that individuals with NSSI exhibit heightened responses to sad emotions (44), while a study on microstate dynamics under negative emotion provides supporting evidence (45). Microstate B is closely associated with bilateral occipital cortices, primarily involving the visual network (46). Abnormalities in emotion recognition implicate basic visual processes (47). During emotional processing, microstate B activity correlates with visual network activation, potentially playing a critical role in the initial perceptual stage of emotional stimuli, particularly when processing emotionally salient visual inputs (48). Therefore, alterations in microstate B may be explained by visual stimulation from video-viewing in the experimental protocol. We propose that microstate A could serve as a biomarker for aberrant pain perception in MDD+NSSI patients.

Compared to the HC group, the reduction of microstate B in the MDD group contradicts some prior findings (49, 50). Patients with mood disorders typically exhibit increased microstate B (51). However, A study observed decreased microstate B in patients with definitive therapeutic responses (52), suggesting that reduced microstate B might predict treatment efficacy. This partially explains the decreased microstate B in the MDD group in our study. Previous conclusions on microstate B are inconsistent, likely due to differences in depressive subtypes and treatments across studies. Future researchers can explore this further. The increased occurrence of microstate A in the MDD group compared to HC has been consistently validated. Several studies support our findings regarding the increased occurrence of microstate A and elevated D→A transition rates in the MDD group (53–56).

It is notable that significant differences in microstate dynamics metrics were not found between the MDD group and the MDD+NSSI group. Under identical emotional arousal conditions, EEG microstate differences between HC and MDD groups primarily stem from MDD pathology. The MDD+NSSI subgroup inherently belongs to the MDD spectrum, and their microstate alterations should theoretically align with the MDD group. In fact, this consistency does exist. Unexpectedly, no significant differences were found between the MDD group and the MDD+NSSI group. A nociceptive discrimination task performance and a resting-state EEG study also show similar findings (8, 53). However, studies examining the HPA axis response to pain stimuli in adolescents with NSSI have found enhanced N2 amplitude in both the spatial resolution paradigm and the mental arithmetic paradigm in the NSSI group (9). The contradictory results may be attributed to differences in paradigms and the inherent heterogeneity of the population. The NSSI adolescent participants in our study exhibited a wide range of self-injury severity, which was generally mild. This may have directly contributed to the non-significant differences observed between groups. Future studies should exercise caution in selecting participants to better test the study hypothesis.

To explore the lack of significant differences in microstates between the MDD group and the MDD+NSSI group, the data were further analyzed. An interesting phenomenon was observed: during pain experience, all microstate dynamics metrics with significant group differences related to the MDD group showed the same trend. That is, compared to the HC group, both the MDD group and the MDD+NSSI group showed the same trend of change, but the MDD+NSSI group had a smaller change magnitude than the MDD group. The results of the correlation analysis can explain this change. The duration and coverage of microstate A were negatively correlated with pain tolerance and positively correlated with FPS-R scores. The MDD+NSSI group exhibited reduced pain perception, characterized by increased pain tolerance, increased pain threshold, and decreased FPS-R scores. That is, compared to the MDD group, the duration and coverage of microstate A were reduced in the MDD+NSSI group. This is consistent with the actual analysis results of EEG microstate dynamics. Hypoalgesia may primarily arise from intracortical processing alterations analogous to those observed in meditative states (8). ACC is critically involved in both emotional regulation and pain processing pathways (32). The insula not only participates in pain modulation but also shows robust associations with affective responses to nociceptive stimuli (57, 58). ACC interacts with regions such as the insula through extensive cortical and subcortical connections, supporting its role in emotional and cognitive functions (59, 60). That is, during NSSI behavior under negative emotional states, the anterior cingulate cortex and insula are closely involved in brain activity and interact to regulate emotions and pain. NSSI behavior is reinforced by providing immediate emotional relief, which increases the frequency of self-injury and thus creates a vicious cycle. In patients with NSSI, the activation of the insula and ACC during pain stimuli is significantly lower than in healthy controls, indicating blunted pain perception. Chronic NSSI behavior leads to abnormal regulation, resulting in reduced pain perception. This altered response may be associated with abnormalities in the endogenous opioid system, such as beta-endorphin release (61). The inhibitory effect of NSSI behavior on Negative emotion is likely mediated through this pathway (62, 63). Therefore, we hypothesize that recurrent NSSI behaviors in MDD patients with NSSI may induce certain alterations in their anterior cingulate cortex or insula regions, leading to diminished magnitude of EEG microstate dynamics during pain processing compared to the MDD group. These changes in large-scale brain network dynamics likely constitute the neurophysiological basis for aberrant pain perception following Negative emotion in NSSI patients.

In summary, patients with MDD who exhibit NSSI behavior show significant abnormalities in pain perception behavior following negative emotions. Moreover, the changes in pain intensity and pain tolerance are significantly correlated with certain microstate dynamics indices. During the process of abnormal changes in pain perception, the way patients cognize and process pain and emotions is of vital importance. We suggest that in psychotherapy, emphasis should be placed on correcting NSSI patients’ cognition of self-harm and their behavior of alleviating emotions through self-harm. Additionally, it can be considered to target the anterior cingulate cortex and insula for Transcranial Magnetic Stimulation (TMS) treatment. Future research could compare various methods of evoking emotions (e.g., pictures, texts) and pain (e.g., laser-evoked potentials, thermal pain, pressure pain). Considering individual differences in pain sensitivity, it is also important to study individuals who do and do not perceive pain in response to the same stimulus. Additionally, given the close association between NSSI behavior and cognitive function, the relationship between reduced pain perception and cognitive function also deserves investigation.

### 4.1 Limitations

First, the cross-sectional nature of the study limits its ability to argue the long-term effects of NSSI behavior on pain perception and microstates. Future prospective longitudinal studies are needed. Second, the NSSI adolescents included in the study had heterogeneous illness durations and varied self-injury severity, which was generally mild. While we identified significant changes in pain-related and microstate metrics under negative emotions compared to neutral emotions in individuals with self-injury, no significant differences were observed between the MDD and MDD+NSSI groups. Future studies could screen participants based on self-injury severity or select participants based on pain perception. Third, the small sample size limits the generalizability of the study results. Future research should employ larger and more diverse samples to verify and expand upon the current findings. Fourth, due to the emotional instability of patients with MDD, the emotional induction materials used in the study were presented in a fixed order rather than a random order. This arrangement may have led to potential order effects. Future research should randomize the presentation order of the emotional induction materials.

## 5 Conclusion

In this study, we observed significant abnormalities in pain perception behaviors following negative emotional stimuli among MDD patients with NSSI. Notably, the altered pain intensity and pain tolerance were significantly correlated with specific microstate dynamics indicators, suggesting that these changes in large-scale brain network dynamics may serve as the neurophysiological basis for aberrant pain perception post-negative emotion in the MDD+NSSI group. These findings provide novel insights into the investigation of NSSI behaviors in adolescents with MDD. We encourage further studies to verify our findings and hope to incorporate neuroimaging research in the future.

## Data Availability

The raw data supporting the conclusions of this article will be made available by the authors, without undue reservation.
