# Correction: Study of pain perception under negative emotions and its large-scale brain network dynamics in adolescents with non-suicidal self-injury

**DOI:** 10.3389/fpsyt.2025.1686418

**Published:** 2025-09-04

**Authors:** Yang Zhou, Dongdong Zhou, Zhengyong Zhang, Huiyu Jia, Fang Chen, Liuyi Ran, Xiaorong Chen, Liyang Wan, Yijia Wang, Wo Wang

**Affiliations:** Mental Health Center, University-Town Hospital of Chongqing Medical University, Chongqing, China

**Keywords:** non-suicidal self-injury (NSSI), adolescent, cold pressor test (CPT), EEG microstates, large-scale brain network dynamics

There was an error in the **Funding** statement. An incorrect grant number was provided for Chongqing Science and Health Joint Project. The correct grant number is “(2024MSXM139)”.

The original version of this article has been updated.

